# A large scale comparative genomic analysis reveals insertion sites for newly acquired genomic islands in bacterial genomes

**DOI:** 10.1186/1471-2180-11-135

**Published:** 2011-06-15

**Authors:** Pengcheng Du, Yinxue Yang, Haiying Wang, Di Liu, George F Gao, Chen Chen

**Affiliations:** 1 National Institute for Communicable Disease Control and Prevention, Center for Disease Control and Prevention/State Key Laboratory for Infectious Disease Prevention and Control, Beijing 102206, China; 2 Affiliated Hospital of Ningxia Medical University, Ningxia 750001, China; 3 CAS Key Laboratory of Pathogenic Microbiology and Immunology, Institute of Microbiology, Chinese Academy of Science, Beijing, China; 4 Network Information Center, Institute of Microbiology, Chinese Academy of Sciences, Beijing, China; 5 Beijing Institutes of Life Science, Chinese Academy of Sciences, Beijing, China

**Keywords:** switch sites of GC-skew, genomic island, evolution

## Abstract

**Background:**

Bacterial virulence enhancement and drug resistance are major threats to public health worldwide. Interestingly, newly acquired genomic islands (GIs) from horizontal transfer between different bacteria strains were found in *Vibrio cholerae, Streptococcus suis*, and *Mycobacterium tuberculosis*, which caused outbreak of epidemic diseases in recently years.

**Results:**

Using a large-scale comparative genomic analysis of 1088 complete genomes from all available bacteria (1009) and Archaea (79), we found that newly acquired GIs are often anchored around switch sites of GC-skew (sGCS). After calculating correlations between relative genomic distances of genomic islands to sGCSs and the evolutionary distances of the genomic islands themselves, we found that newly acquired genomic islands are closer to sGCSs than the old ones, indicating that regions around sGCSs are hotspots for genomic island insertion.

**Conclusions:**

Based on our results, we believe that genomic regions near sGCSs are hotspots for horizontal transfer of genomic islands, which may significantly affect key properties of epidemic disease-causing pathogens, such as virulence and adaption to new environments.

## Background

DNA strands in most prokaryotic genomes often experience strand-biased spontaneous mutations, especially in protein coding regions, which occur preferentially in the leading strand during DNA replication [1,2]. It has been found that the directions of GC skew often change at flanking regions around bacterial replication origins [3-8]. Therefore, strand compositional asymmetry is commonly used to identify locations of bacterial replication origins [3-7]. To date, strand asymmetry has been widely studied with GC-skew analysis by calculating [G-C]/[G+C] in the chromosome or protein coding regions [9,10]., Additionally, bacterial genomes share many other asymmetric features, such as gene density, strand direction, purine content in genes, and codon usage [11]. Most interestingly, many bacteria with strong evolution selection pressure display extremely biased GC skew [12]. Correspondingly, GC-skew analysis is often utilized as a method for measuring selection pressure of different genome replication machineries [7,12,13]

While mutations generated during replication are an important source of bacterial compositional asymmetry, horizontal acquisition of foreign DNAs, known as genomic islands (GIs), also plays an important role. GIs can affect compositional bias, by changing the GC content, introducing new codon usage bias, and altering dinucleotide signature. GIs encode many different functions and are thought to have played a major role in the microbial evolution of specific host-recognition, symbiosis, pathogenesis, and virulence [14,15].

In genomes of human pathogens, pathogenicity islands (PAIs) are the most significant GIs. They often contain functional genes related to drug resistance, virulence, and metabolism [16-18]. One such example, *Vibrio cholerae *pathogenicity island-2 (VPI-2) was found to encode restriction modification systems (*hsdR *and *hsdM*), genes required for the utilization of amino sugars (*nan-nag *region), and a neuraminidase gene [19,20]. These results suggest that VPI-2 might be an essential region for pathogen survival in different ecological environments and hence increase virulence [19]. It is thought that VPI-2 might have been acquired by *V. cholerae *from a recent horizontal transfer [19,20]. Similarly, 89K genome island might have been the major factor for *Streptococcus suis *outbreaks, such as the one in China in 2005 [21]. Therefore accurate identification of GI regions is of utmost importance.

**Figure 1:**
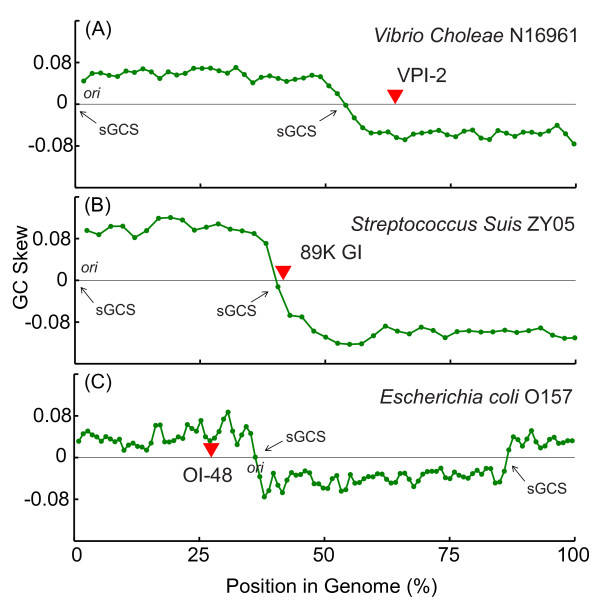
**Relation between sGCSs and GIs**. Three genome islands in *Vibrio Choleae *N16961, *Streptococcus Suis *ZY05 and *Escherichia coli *O157 were plotted with sGCSs.

sGCS, switch sites of GC-skew, arises when the G/C bias on the chromosome abruptly changes [22]. Because GIs come from other bacteria probably with a different G/C bias, the GIs can introduce new switch sites and should theoretically be located adjacent to them. However, the relationships between switch sites and GIs have not been previously investigated on metagenomics scale. To illustrate the relationship between sGCSs and GIs, we used *V. cholerae, Streptococcus suis *and *Escheichia coli *as an example (Figure 1). In this study, we focus on the strategies for identifying GIs and switch sites of GC-skew (sGCS) and propose a new term, putative GI (pGI), to denote abnormal G/C loci as GI insertion hotspots in bacterial genomes. With this new terminology, we developed a novel comparative genomic algorithm, based on genomic and evolutionary distance between pGIs and sGCSs, to identify functional attributes that predict the potential locations for GI insertions. Furthermore, we provided strategies for identifying new GIs in different groups of bacteria, which might be potential pathogens for infectious diseases.

## Methods

### 2.1 Complete genomic sequences and their bias features

Complete bacterial genomes and annotation files were downloaded from the NCBI database ftp://ftp.ncbi.nih.gov/genomes/Bacteria/. The features of the genomes (e.g., organism names, lineages, chromosome topologies, *dnaA *gene locations, GC contents, and GC coordinates) were used in the comparative genomic analysis. Genome bias switch signals were detected by signals of the GC skews along the genomes, calculated by [G - C]/[G + C] with window sizes of 100-kb and steps of 50-kb. Here, sGCSs are defined as the sites at the cross point of GC skew and the average GC content.

### 2.2 GIs and their physical distances

For each genome, we calculated GC content with a window size of 2000-bp and a step size of 1000-bp. In our analysis, pGIs were usually > 5 kb. As controls, Pathogenity Island (PAI), PAI-like sequences overlapping with GIs (candidate PAIs, cPAIs), and PAI-like sequences not overlapping GIs (non-probable PAIs, nPAIs) data were downloaded from the PAI database http://www.gem.re.kr/.

### 2.3 Genomic and evolutionary distances

The genomic distances between GIs and sGCSs were calculated using their genomic coordinates. For each GI, the distance to the sGCSs was determined by the nearest sGCS. To compare genomic distances between different species, instead of using physical distances, we obtained relative distances by dividing them with the length of each genome. This way, relative distances in different genomes are on the same scale (0 to 1) and are thus mutually comparable. GI homologues were obtained by searching evolutionarily highly-correlated bacterial genomes. GIs found in at least two strains were selected for analysis. For each pair, the BLASTN algorithm was used to evaluate their similarity. GIs with ≥ 80% overlap to each other were considered pairs of homologues. Evolutionary distance between each pair was obtained by the sequence similarity distance in the HKY85 model using PAUP [23,24]. The matrix of distances was parsed to obtain a list of evolutionary distances. Next, correlations between evolutionary distances between homologous GIs and their corresponding genomic distances were calculated with R. A phylogenic tree was also constructed via the neighbor joining method using PAUP.

## Results

### 3.1 Identifying special features in bacterial genomes: switch signals of GC skews and GIs

The dataset used for this study includes 1090 bacterial chromosomes (from 1009 bacterial species) as samples and 83 chromosomes (from 79 archaeal species) as controls. In a previous study, sGCSs were used to predict origins (*ori*) or termini (*ter*) of replication, as well as gene density and purine excess (C+T-G-A) [5]. It was hypothesized that sGCSs may be important signals for genome bias. In this study, we investigated sGCSs for specific GC content-related genomic features, using 2-kb sliding windows with 1-kb steps along the various genomes. We found that most of the bacteria, such as *Firmicutes*, *Proteobacteria*, and *Bacteroidetes*, contain much fewer sGCSs in their genomes compared to archaea (Table 1). For further comparison, we counted the number of bacteria and Archaea with different numbers of sGCSs (i.e., 2, 4-8, and ≥ 10, Table 1). In the bacteria group, most genomes contain less than eight sGCSs and show a simplified switch model of compositional bias (e.g., *Bacteroidetes *(24/25, 96%) and *Firmicutes *(188/188, 100%)) (Table 1). However, in ancient bacterial genomes, the number of sGCSs is seldom fewer than eight. For example, six of seven *Aquificae *strains have more than eight sGCSs, while 53% of *Actinobacteria *and 44% of *Cyanobacteria *have more than eight (see Table 1).

**Table 1:** Distribution of sGCSs in different phyla.

Taxon	Phylum	# of chromosomes	# of sGCSs			Percentage of sGCSs # < = 8	Average GC+/- SD (%)*	Average Length +/- SD (kb)^$^
			2	4-8	> = 10			
Archaea	*Crenarchaeota*	23	0	5	18	21.74%	44.39 +/- 9.66	2188.85 +/- 506.62
	*Euryarchaeota*	57	7	13	37	35.09%	46.31 +/- 12.66	2211.67 +/- 1034.73
	*Korarchaeota*	1	0	0	1	0.00%	49.75 +/- 0.00	1590.76 +/- 0.00
	*Nanoarchaeota*	1	0	1	0	100.00%	31.60 +/- 0.00	490.88 +/- 0.00
	*Thaumarchaeota*	1	0	0	1	0.00%	33.90 +/- 0.00	1645.26 +/- 0.00

Bacteria	*Acidobacteria*	3	0	0	3	0.00%	60.13 +/- 1.64	6581.12 +/- 3028.39
	*Actinobacteria*	92	20	23	49	46.74%	65.08 +/- 7.01	4563.76 +/- 2248.12
	*Aquificae*	7	0	1	6	14.29%	38.82 +/- 5.91	1680.59 +/- 161.52
	*Bacteroidetes*	29	14	14	1	96.55%	41.95 +/- 11.91	3653.46 +/- 2340.45
	*Chlamydiae*	15	14	1	0	100.00%	40.25 +/- 1.67	1209.16 +/- 343.03
	*Chlorobi*	11	8	3	0	100.00%	50.64 +/- 4.40	2618.73 +/- 417.30
	*Chloroflexi*	14	5	4	5	64.29%	55.78 +/- 7.93	3290.10 +/- 2063.61
	*Cyanobacteria*	41	9	14	18	56.10%	44.76 +/- 10.19	3185.53 +/- 2028.34
	*Deferribacteres*	2	2	0	0	100.00%	36.87 +/- 8.07	2728.23 +/- 698.40
	*Deinococcus-Thermus*	7	3	3	1	85.71%	66.54 +/- 2.43	2170.02 +/- 900.69
	*Dictyoglomi*	2	2	0	0	100.00%	34.66 +/- 0.02	1907.77 +/- 73.84
	*Elusimicrobia*	2	2	0	0	100.00%	38.13 +/- 2.96	1384.71 +/- 366.07
	*Fibrobacteres*	1	1	0	0	100.00%	47.74 +/- 0.00	3842.64 +/- 0.00
	*Firmicutes*	200	198	2	0	100.00%	38.54 +/- 6.93	3081.76 +/- 1184.70
	*Fusobacteria*	4	2	2	0	100.00%	28.83 +/- 3.56	2680.38 +/- 1205.57
	*Gemmatimonadetes*	1	0	1	0	100.00%	64.17 +/- 0.00	4636.96 +/- 0.00
	*Nitrospirae*	1	0	0	1	0.00%	33.91 +/- 0.00	2003.80 +/- 0.00
	*Planctomycetes*	2	1	1	0	100.00%	56.21 +/- 1.74	6670.89 +/- 671.31
	*Proteobacteria*	586	369	155	62	89.42%	53.12 +/- 12.12	3516.36 +/- 1661.41
	*Spirochaetes*	24	21	3	0	100.00%	35.65 +/- 7.38	1680.71 +/- 1445.58
	*Synergistetes*	2	2	0	0	100.00%	54.16 +/- 12.43	1914.53 +/- 93.42
	*Tenericutes*	27	12	15	0	100.00%	27.98 +/- 3.40	892.61 +/- 204.62
	*Thermobaculum*	2	1	1	0	100.00%	56.02 +/- 11.51	1550.79 +/- 673.39
	*Thermotogae*	11	0	6	5	54.55%	40.19 +/- 6.51	1976.74 +/- 160.46
	*Verrucomicrobia*	4	3	1	0	100.00%	55.24 +/- 8.47	3664.91 +/- 1649.61

Total		1173	696	269	208	82.27%		

* Average GC content and standard deviations (SD) were calculated according to the different strains in the phylum.

^$^Average length was calculated by averaging the complete genome length in the phylum.

The acquisition of foreign DNA may modify compositional bias, and GC content change is a predominant outcome of this process. Another outcome of foreign DNA insertion is the appearance of GIs, which may change the virulence or function of the host strain (Figure 1D). In this study, we calculated GC content deviations for all the bacterial genomes. Then, we searched the genomic sequence for GIs by identifying the genomic segments with GC contents significantly different from the mean value of the genome (i.e., greater than three times the standard deviation). From all of the genomes analyzed, 20,541 GIs were detected, according to the above criteria, with lengths from 2 to 80 kb, depending on the size of the sliding window used.

### 3.2 GIs are located next to sGCSs

**Figure 2:**
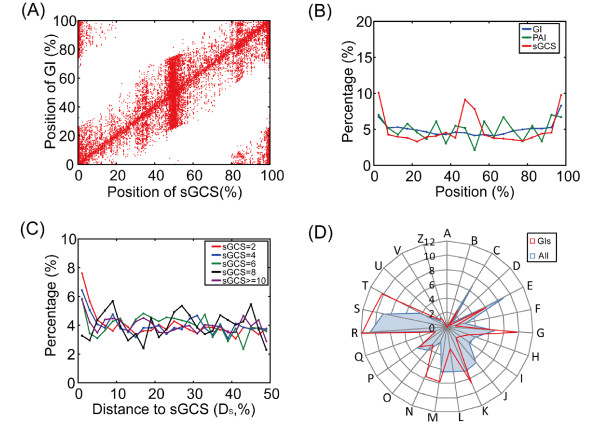
**Distribution of GI, sGCS, and PAIs in the genome**. (A) Scatter plot of the positions of GIs vs. sGCSs. For each genome, we coupled the positions of sGCSs and GIs. (B) Distribution of sGCSs, GIs, and PAIs in the genome. (C) Frequency of Ds along the genome with different sGCSs groups. (D) Gene classification according to COG functions in GIs (red) and all of the genomes. For each category: A, RNA processing and modification; B, Chromatin structure and dynamics; C, Energy production and conversion; D, Cell cycle control, cell division, chromosome partitioning; E, Amino acid transport and metabolism; F, Nucleotide transport and metabolism; G, Carbohydrate transport and metabolism; H, Coenzyme transport and metabolism; I, Lipid transport and metabolism; J, Translation, ribosomal structure, and biogenesis; K, Transcription; L, Replication, recombination, and repair; M, Cell wall/membrane/envelope biogenesis; N, Cell motility; O, Posttranslational modification, protein turnover, chaperones; P, Inorganic ion transport and metabolism; Q, Secondary metabolites biosynthesis, transport and catabolism; R, General function prediction only; S, Function unknown; T, Signal transduction mechanisms; U, Intracellular trafficking, secretion, and vesicular transport; V, Defense mechanisms; and W, Cytoskeleton.

Bacterial genomes exhibit strong sGCSs signals, which is easy to understand because the genomes of different strains often share one replicon (Figure 2 AB). For a better comparison, we aligned all the genomes at the *ori*, and calculated relative genomic positions by dividing them with the length of each genome. sGCSs and pGIs were then plotted according to their relative genomic positions. When aligned at the origin and marked with relative distances, the genomes had an overrepresentation of sGCSs at 1/3, 1/2, and 3/4 marks. (Figure 2 AB). Furthermore, we found that aside from their special distribution (Figure 2 A), sGCSs are closely correlated with GIs. These GIs are thought to have come from lateral gene transfer (LGT) events between different species but not from vertical inheritance due to their different genomic features. Based on the correlation between sGCSs and GIs, we suspect that sGCS regions are hotspots for horizontal DNA transfer in bacterial genomes,

The diversified frequency of sGCSs and variation of GC skews in different genomes usually indicate different replication mechanisms. To investigate the relationship between sGCSs frequency and replication mechanisms, we separated the genomes in the study into several groups according to their sGCS numbers. For example, in most typical *Firmicutes *(i.e., gram-positive bacteria), such as *S. suis*, replicons often display specific patterns and can therefore be easily detected in the genome. *Firmicutes' *sGCSs are most often located at the replication *ori/ter *and the middle of the genomes. Therefore, the number of sGCSs is usually two. In some strains used in industry, such as *Streptomyces avermitilis*, the number of sGCSs is often greater than two because these strains employ different replication mechanisms. Furthermore, in bacteria such as *Yersinia pestis *KIM and *Y. pestis *91001, sGCS distributions vary significantly due to large scale genome rearrangements, duplications, and insertions. Notably, we found that the appearance of GIs near sGCSs is not impacted by these replication mechanisms and rearrangements. After categorizing the genomes according to their sGCS numbers, we found that for all categories, GIs are highly enriched in the sGCS flanking regions (Figure 2C).

Recently acquired GIs were found in a significant number of pathogen isolates [21,25]. Example of such PAIs are VSP I and II in *V. cholerae*, which are only found in the *Vibrio *seventh pandemic. LEE, a well-known GI in *Escherichia coli *O157, encodes structural, accessory, effector, and regulatory molecules and is located near to *ter *sites [25]. An additional 87-kb O island 48 (OI-48) is found in O157:H7 strains, EDL933, and Sakai, which is associated with tellurite-resistance. Our analysis successfully identified these GIs, demonstrating the validity of our approach. Another example of this type of recently acquired island is a 89-kb genome fragment in *S. suis *that contains zeta-toxin, a two-component signal transduction system, and three ABC transporter cassettes [21]. Again, these islands with genes related to the toxins and infectivity of pathogens are all located near sGCSs, indicating the correlations between GIs and sGCSs.

### 3.3 Based on a phylogenetic analysis, newer GIs are more likely to occur closer to sGCSs

**Figure 3:**
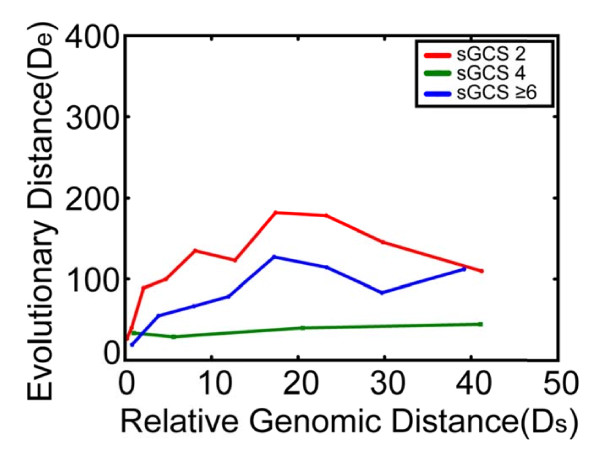
**Correlation between GI evolutionary distance and relative genomic distance**. For each GI group, relative genomic distance and evolutionary distance were calculated. Along the relative genomic distance, average evolutionary distance were calculated. Average evolutionary distance was then plotted against relative genomic distance to reveal the correlation between relative genomic distance and evolutionary distance.

To identify the origins of the GIs examined, we clustered the 14,921 pGIs from the 1009 bacterial genomes into 158 groups and then conducted a large-scale phylogenetic analysis. Our analysis revealed that the evolutionary distances of GIs are highly correlated with their genomic positions. Two distances, the physical distance between a pGI to the closest sGCS (D_s_) and the evolutionary distance (D*_e_*) between two homologus pGI, were calculated. For each homologue group, we plotted these two distances. To study the correlation between D_s _and D*_e_*, we performed regression analysis on the two distances (Figure 3). For the genomes with two sGCSs, we saw a clear pattern. The plot of D_s _vs. D_e _reveals a positive correlation (correlation = 0.818) in 0-25% genomic regions and a negative correlation (correlation = -0.762) 25-50% regions (Figure 3). These results show that for the pGIs near sGCSs (0-50%), the correlation is statistically significant. The results agree with recent acquisitions of these genomic islands, which were horizontally transferred into the susceptible regions of the genomes recently and are therefore closer to sGCSs. However, when the distance of a pGI to the nearest sGCS is greater than 25% of the distance in the genomes with two sGCSs, the correlation is reversed, (i.e., the evolutionary distance is reduced with the increasing of the physical distance from the sGCS). This observation indicates that when GIs were inserted in genomic regions far from sGCSs, positive correlations between physical distances and evolutionary distances no longer hold. However, we did not find clear patterns for genomes with more than two sGCSs.

The phylogenic analysis of all of the GI groups also suggests the correlation between D_s _and D_e. _For example, the well-known toxin co-regulated pilus (TCP) GI, found in four strains (N16961, MJ-1236, M66-2, and O395) is located at 43.40, 43.58, 44.64, and 49.07% in the genomes, respectively. We used N16961 as a standard for normalization and obtained evolutionary distances for the other three strains (0, 0, 0.00002, and 0.0003). Again, we observed a strong correlation between D_s _and D_e_, indicating that in highly conserved genomes, the physical distances of GIs to sGCSs are highly correlated with the evolutionary distances between them.

## Discussion

Virulence properties of particular strains within a species are often associated with the presence of specific horizontally acquired genetic elements [21]. The Human Haplotype Project has identified the vast majority of conserved genome fragments, which separate the human genome into numerous blocks [26,27]. Recently, a similar study on *Y. pestis *revealed that the mosaic structure of these blocks also exists in bacterial genomes [28]. The boundaries of the blocks are thought to be hotspots of recombination and insertion. For example, the major histocompatibility complex (MHC) is located between such blocks [29]. Our study sheds light on the hotspots in genomes for GI insertion using a large scale comparative genomic method. Our results suggest that GIs are likely to be inserted at the block boundaries of genomes of bacteria and other microbes, and sGCSs in these genomes are common separation spots for such blocks.

Via a phylogenetic analysis of each pGI and its homologues, we obtained the evolutionary distance for each pair of homologous pGIs. After studying the correlation between D_s _and D_e_, we found that they are positively correlated in regions closer to sGCSs (0-25%), while the correlation is reversed in more distal regions (25 - 50%). The turning point is near 25% region for geomes with two sGCSs. The mechanism underlying this phenomenon is currently unclear but may be caused by genomic rearrangements or deletions.

In human pathogens, many PAIs are found in GIs, such as VSP I and II in *V. cholerae*. However, generally speaking, PAIs and GIs refer to different genomic features. On the one hand, PAIs are sometimes evaluated by sequence similarity in other species, and these PAIs do not display abnormal GC content. Additionally, not all GIs are associated with pathogens. For example, in *E. coli *CTF073, none of the four abnormal GC content regions matches PAIs. These PAIs are different from typical PAIs due to special genomic rearrangement mechanisms. According to our observations, only laterally transferred GIs and newly acquired GIs are found near sGCSs. Notably, these types of horizontally transferred GIs were discovered in recent emerging infectious diseases and proven to enhance virulence or adaption of such strains [21,30]. Therefore, GIs are of great importance in revealing the mechanisms of certain epidemic diseases. From the observation that GIs are likely to be inserted at genomic block boundaries, we propose that important virulence factors, which are associated with the outbreaks of many common diseases and/or enhanced virulence can be found near sGCSs.

## Conclusion

In this study, in order to do a large scale study on the properties of genomic island, we used 1090 bacterial chromosomes (from 1009 bacterial species) as samples and 83 chromosomes (from 79 archaeal) as controls and separated them into three groups (sCGSs < = 2; 4 < = sCGSs < = 8; sCGSs > = 10) according to the number sCGSs. Interestingly, most of bacteria genomes contain less than 8 sCGSs, while archaeal genomes often contain more than 8 sCGSs. We then searched the genomic sequence for GIs by identifying the genomic segments with GC contents significantly different from the mean value of the genome and detected 20,541 GIs. We separated the GIs into different homolog groups and studied the correlation between relative genomic distance and evolution distance and found that sGCS regions are hotspots for horizontal DNA transfer in bacterial genomes. Since this is the first time for such an important property to be revealed by a large scale comparative genomic method, we believe our finding is of great importance for predicting both genomic island and their insertion sites.

## Abbreviations

sGCS: switch site of genome GC skew; GI: genomic island; PAI: pathogenicity islands; pGI: putative genomic island.

## Competing interests

The authors declare that they have no competing interests.

## Authors' contributions

PD and HW carried out genome island analyses. DL contributed to database and data organization. GFG and CC designed the project and editing of the manuscript. YY and CC wrote the final manuscripts. All authors read and approved the final manuscript. The authors declare no conflict of interest.
